# Differential Analysis of Hypertension-Associated Intestinal Microbiota

**DOI:** 10.7150/ijms.65972

**Published:** 2021-10-08

**Authors:** Xie Dan, Zhang Mushi, Wang Baili, Lin Han, Wu Enqi, Zhao Huanhu, Li Shuchun

**Affiliations:** 1 School of Pharmacy, Minzu University of China, Key Laboratory of Ethnomedicine (Minzu University of China), Ministry of education, Beijing 100081, P.R. China; 2 School Hospital, Minzu University of China, Beijing 100081, China

In our paper 1, the first sentence of the result should be corrected as “A total of 67 cases of HTN and 62 cases of normal BP were included in this study”, and Table 1 should be corrected as follows.

## Figures and Tables

**Table 1:** Characteristics of subjects

	Normal pressure	Blood Hypertension	P-value
Sample	62	67	
Gender (% Female)	62 (53.23%)	67 (61.19%)	0.360
Age, year	69.322 ± 10.613	69.492 ± 9.630	0.509
SBP, mmHg	122.935 ± 6.902	153.298 ± 14.917	0.000*
DBP, mmHg	76.209 ± 6.902	84.313 ± 10.739	0.000*
BMI, kg/m^2^	26.089 ± 3.112	25.051 ± 4.436	0.169
WHR	0.864 ± 0.065	0.853 ± 0.130	0.645
GLU, mmol/L	6.729 ± 1.956	6.030 ± 1.176	0.059
CHO, mmol/L	5.249 ± 1.022	5.093 ± 1.062	0.443
HDL, mmol/L	1.403 ± 50.827	1.441 ± 0.346	0.496
LDL, mmol/L	1.986 ± 1.250	3.182 ± 0.865	0.322
TG, mmol/L	1.986 ± 1.250	1.812 ± 1.206	0.336
BUN, mmol/L	5.373 ± 1.285	5.739 ± 1.418	0.181
UA, μmol/L	344.709 ± 89.964	346.224 ± 76.830	0.386
HCY, μmol/L	13.940 ± 6.080	13.150 ± 3.620	0.607

The data on age, SBP, DBP, BMI, WHR, GLU, CHO, HDL, LDL, TG, BUN, UA, and HCY are expressed as the mean ± SD. P-values for gender, age, SBP, DBP, BMI, WHR, GLU, CHO, HDL, LDL, TG, BUN, UA, and HCY were calculated using Student's *t*-test. *P < 0.05. SBP: systolic blood pressure, DBP: diastolic blood pressure, BMI: body mass index, WHR: waist-to-hip ratio; GLU: glucosuria; HDL: high density lipoprotein; LDL: low density lipoprotein; TG: total triglyceride, CHO: total cholesterol; BUN blood urea nitrogen; UA: uric acid; HCY: homocysteine. *P < 0.05.
